# Idiosyncrasy against Morphine1Abstracted for the Journal by Dr. Otto G. Noack, Reading, Pa.

**Published:** 1896-08

**Authors:** 


Idiosyncrasy against Morphine.[1] Frœher has informed us that cattle show after injections of morphine, when it is used to quiet in cases of prolapsus uteri, disagreeable excitement and augmented pressing sometimes. Prof. Albrecht has never noted any good results of morphine in such cases, and saw a case a short time ago affirming Frœher's observations. A fat cow, six years old, weighing 1200 pounds, was put in the obstetrical section of the Veterinary Academy for delivery. On the 7th of June premature parturition was suspected, and occurred on the 8th of June. The afterbirth, although only taken fifty-six hours afterward, was so decomposed that fragments of it stayed in the uterus. On the next day violent

[1] Abstracted for the Journal by Dr. Otto G. Noack, Reading, Pa.

straining set in; the cow received a hypodermic injection of morphine, 1 gramme, at 12 o'clock noon. About twenty minutes later the following symptoms appeared: excitement, violent bellowing, and then moaning, continuous bellowing, putting down and lifting up the diagonal feet at the same time. Temperature was 40.5° C., head warm, pupils enlarged, pulse 90–100, respiration 30–35 in a minute. The quantity of milk during this time was diminished; the quantity of cream was not half as much as prior to attack, and did not increase afterward. The eyes of the animal were covered, ice was put on the head, and 50 grammes of chloral hydrate were given. About 11 o'clock P.M. the cow was quiet, and the straining had disappeared.

In Japan most of the horses are shod with straw. Even the clumsiest of cart-horses wear straw shoes, which, in their cases, are tied around the ankle with straw rope, and are made of the ordinary rice-straw, braided so as to form a sole for the foot about half an inch thick. These soles cost about a halfpenny a pair. In Iceland horses are shod with sheeps' horn. In the valley of the Upper Oxus the antlers of the mountain deer are used for the same purpose, the shoes being fastened with horn pins. In the Soudan the horses are shod with socks made of camels' skin. In Australia horseshoes are made of cowhide. A German not long ago invented a horseshoe of paper, prepared by saturating with oil, turpentine, and other ingredients. Thin layers of such paper are glued to the hoof till the requisite thickness is attained, and the shoes thus made are durable and impenetrable to moisture.—*Horseshoers' Journal.*

